# Comparison of Cell Viability and Embryoid Body Size of Two Embryonic Stem Cell Lines After Different Exposure Times to Bone Morphogenetic Protein 4

**Published:** 2015-03

**Authors:** Nehleh Zarei Fard, Tahereh Talaei-Khozani, Soghra Bahmanpour, Tahereh Esmaeilpour

**Affiliations:** Laboratory for Stem Cell Research, Department of Anatomical Sciences, School of Medicine, Shiraz University of Medical Sciences, Shiraz, Iran

**Keywords:** Embryonic stem cells, Bone morphogenetic protein 4, Embryoid body

## Abstract

**Background:**

Activation of bone morphogenetic protein 4 (BMP4) signaling pathway in embryonic stem (ES) cells plays an important role in controlling cell proliferation, differentiation, and apoptosis. Adverse effects of BMP4 occur in a time dependent manner; however, little is known about the effect of different time exposure of this growth factor on cell number in culture media. In this study, we investigated the role of two different exposure times to BMP4 in cell viability, embryoid body (EB), size, and cavitation of ES cells.

**Methods:**

Embryonic stem cells (R1 and B1 lines) were released from the feeder cell layers and were cultured using EBs protocol by using the hanging drop method and monolayer culture system. The cells were cultured for 5 days with 100 ng/mL BMP4 from the beginning (++BMP4) or after 48 h (+BMP4) of culture and their cell number were counted by trypan blue staining. The data were analyzed using non-parametric two-tailed Mann-Whitney test. P<0.05 was considered as significant.

**Results:**

In EB culture protocol, cell number significantly decreased in +BMP4 culture condition with greater cavity size compared to the ++BMP4 condition at day 5 (P=0.009). In contrast, in monolayer culture system, there was no significant difference in the cell number between all groups (P=0.91).

**Conclusion:**

The results suggest that short-term exposure of BMP4 is required to promote cavitation in EBs according to lower cell number in +BMP4 condition. Different cell lines showed different behavior in cavitation formation.

## Introduction

Bone morphogenetic protein 4 (BMP4) is a member of the transforming growth factor-beta (TGF-ß) superfamily of secreted proteins. Receptors of BMP4 form heteromeric complexes as tetrameric transmembrane protein. After activation of BMP receptors, receptor-regulated Smads (Smad1, Smad5, or Smad8) are phosphorylated and bound to Smad4 that translocate from the cytoplasm to the nucleus and regulate transcription of target genes.^1^ BMPs are very multifunctional and are implicated in the control of proliferation, differentiation, and cell-fate determination. BMPs also induced apoptosis in diverse developmental stages, including embryo cavitation, brain and eye development, digit morphogenesis and tissue homeostasis.

Embryo cavitation is necessary for the beginning of gastrulation and consequently the following stages of embryogenesis.^2^ It is shown that mutation in BMP4 signaling molecules can lead to embryo death in the early stage of development. Expression of BMP4 genes of extraembryonic ectoderm in pre-implantation embryos lead to the development of extraembryonic endoderm and BMP2 production. BMP4 and 2 promote formation of the proamniotic cavity by induction of apoptotic cell death. These events (embryo cavitation) prepare the embryo for gastrulation and formation of layers.^3^ Notably, it is non-ethical and also difficult to study the effects of various reagents such as BMP4 on programmed cell death pathways and cell survival during embryo cavitation in vivo. An appropriate in vitro model can closely mimic the in vivo system for the investigation of different role of BMP4 in cell proliferation/differentiation/apoptosis is demanding.^1^^,^^2^

Embryonic stem cells are pluripotent cells derived from pre-implantation embryos and are capable to differentiate into all derivatives of the three primary germ layers. Most usually, initiation of cell differentiation performs by spherical aggregation of ES cells into a so-called embryoid body (EB). Embryoid body formation provides an effective method for three-dimensional culture of ES cells for induction of differentiation in vitro.^4^^-^^6^ Central cavity and visceral endoderm formation in EB due to cell death mimic early development of mouse embryo.^7^^-^^9^ Moreover, in vitro differentiation of ES cells can start with a phase known as monolayer culture system. In this approach, the cell-cell contact reduces; therefore, the survival of the growing cells may be affected.^10^

Embryonic stem cell exposure to BMP4 could induce differentiation toward particular cell lineages in a time- and dose-dependent manner.^11^^-^^13^ It has been known that different ES cell lines react to a growth factor in a variety of ways.^14^ Previous reports also showed that effects of BMP4 on proliferation, differentiation and self-renewal in ES cells are depending on the dose of this growth factor added in culture;^15^^,^^16^ however, there are few investigations regarding the effects of prolonged or short BMP4 exposure time in regulating cell proliferation or death.

In the present study, we designed two different exposure times of BMP4 for the assessment of cell number during EB cavitation and monolayer culture systems. The aim of this study was to find if the duration and time of exposure of the stem cells to BMP4 might affect cell population and cavity formation. 

## Materials and Methods

*Embryonic Stem Cell Culture*

R1^17^ and B1^18^ (XY) mouse ES cells with normal karyotype were cultured on mouse embryonic fibroblasts (MEF) as feeder layer at a density of 1.2×10^6^ cells/T25 flasks. The cells were grown in knockout-Dulbecco’s Modified Eagle’s Medium (KO-DMEM; Gibco, Germany) supplemented with 15% ES-qualified FBS (Biowest, France), 1% penicillin/streptomycin (Gibco, Germany), 2 mM L-glutamine (Gibco, UK), 0.1 mM non-essential amino acids (Gibco, UK), 0.1 mM β-mercaptoethanol (Sigma, USA) and 1000 U/mL leukemia inhibitory factor (LIF; Chemicon) at 37^o^C and 5% CO_2_. The cultures were incubated at 37°C in a humidified 5% CO_2_/95% air incubator. The medium was changed every day and cells were passaged every 2-3 days.

*Feeder Layer Preparation*

Mouse embryos at 12.5 or 13.5 days old were removed from uterine horn and head and red organs were removed carefully. Remaining tissues were pushed through a 18 gauge needle to disaggregate the embryos. Suspension was cultured in DMEM (Gibco, Germany) supplemented with 15% FBS, 2 mM L-glutamine, 0.1 mM β-mercaptoethanol, 1% Penicillin/Streptomycin. The cells were harvested at 90% confluence and subcultured in the same medium. At passage 3-5, mitotically inactivated cells with 10 µg/ml mitomycine-C (Sigma, USA) were used as feeder layer.

*Administration of BMP4 and Cell Count*

The two undifferentiated ES cell lines, R1 and B1, were trypsinized and a single cell suspension was transferred into a 10 cm tissue culture plate, pre-coated with 0.1% gelatin and incubated at 37°C with 5% CO_2_ for 1-1:30 h. After these times, the fibroblasts have attached to the plate and floating cells were ES cells. Each ES cell line was collected by centrifugation and aliquoted at 1×10^5^ cells/mL in LIF-free ES cell media containing 12% ES-FBS in the presence or absence of 100 ng/mL of BMP4 (R&D systems). Each aliquot was cultured as EB or monolayer culture system for 5 days, as follows:

*-Embryoid Body Culturing*
In the EB culture method, ES cells were cultured in hanging drops with starting cell density of 2000 cells/20 µL onto the lid of 10 cm petri dishes (Corning) filling with 10 mL sterile distilled water. The EBs formed in a medium with or without BMP4 for 2 days. After 48 h, the cell aggregates were picked up with the pipette and transferred into a 60 mm ultralow attachment plates with fresh LIF-free ES media containing 8% ES-FBS in the presence or absence of 100 ng/mL of BMP4 (++BMP or -BMP4) for an additional 3 days. Besides, other condition of BMP4 exposure was added after 48 h from the beginning of culture (+BMP4). The plates were placed into the incubator undisturbed for 3 days. Finally, three experimental groups were carried out for each ES cell line (figure 1): i) Embryoid body culturing in the presence of BMP4 from the initiation of culturing (++BMP4), ii) Embryoid body culturing in the presence of BMP4 after 48 h of culturing (+BMP4), and  iii) Embryoid body culturing in the absence of BMP4 (–BMP4). 
  **Figure 1:**
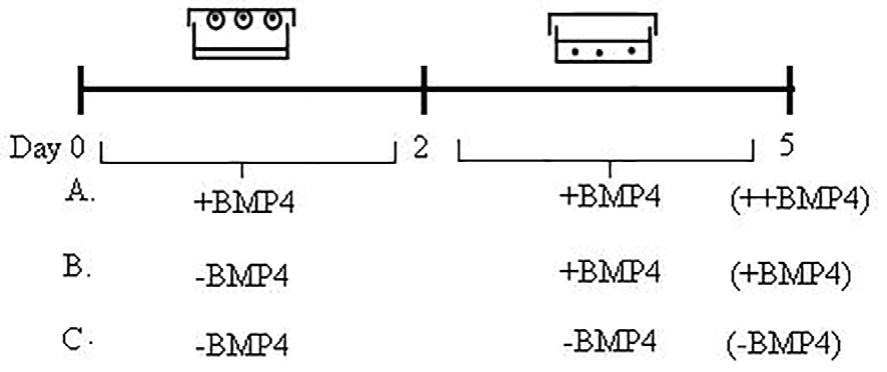
The diagram shows the time schedule of the ES cell differentiation procedures for EB protocols for 5 days. ES cells were aggregated in hanging drop culture for 2 days before transfer in suspension culture. The cell culturing was performed (A) in the presence of BMP4 from the initiation of culture and named as ++BMP4 condition, (B) in the presence of BMP4 after 2 days of culture and named as +BMP4 condition or (C) in the absence of BMP4 and named as –BMP4 condition.
  In B1 ES cell line, in addition to the above protocol, an additional culture with 15000 cells/30 µL with or without BMP4 (+BMP4 and -BMP4) conditions was incubated in hanging drops.
*-Monolayer Culturing*
In monolayer culture system, 3×10^4^cells in a medium with or without BMP4 were transferred into each well of a 24–well plate for 48 h. After 48 h the media were exchanged completely with fresh LIF-free ES media containing 8% ES-FBS in the presence or absence of 100 ng/mL of BMP4 (++BMP or -BMP4) for an additional 3 days. Besides, other condition of BMP4 exposure was added after 48 h from the beginning of culture (+BMP4). The plates were placed into the incubator undisturbed for 3 days. According to the experimental design, three groups were conducted for each ES cell line: i) Monolayer culturing in the presence of BMP4 from the initiation of culturing (++BMP4), ii) Monolayer body culturing in the presence of BMP4 after 48 h of culturing (+BMP4), and iii) Monolayer culturing in the absence of BMP4 (–BMP4). 

*Cell Count*

On days 2 and 5, EB and monolayer cultures were dissociated into a single cell suspension with trypsin/EDTA. The cells were counted and their viability was assessed by trypan blue staining. 

*Morphological Assessment*

The morphological changes during in-vitro culture were recorded every day under the inverted microscope (OLYMPUS) equipped with a Nikon DXM-1200C digital camera with the same light intensity and magnification. For each EB, according to three experimental groups separately, 4 diameters were measured with a calibrated ruler. The ruler was calibrated with an eyepiece. The volume of each EB was estimated using sphere volume formulation (r^ 3 ^π4/3). The exclusion criterion was the smallest and the largest EBs.

*Statistical Analysis*

Each data is presented as mean±SD with five repeats in each experiment. Statistical significance was determined by non-parametric Kruskal-Wallis H test and Mann-Whitney test. All data were analyzed in SPSS 16 software for Windows (SPSS, Chicago, IL, USA). The graphs were plotted with Prism v.5 software (GraphPad Software, San Diego, CA, USA). P value less than 0.05 were considered as significant. 

## Results

**Figure 2:**
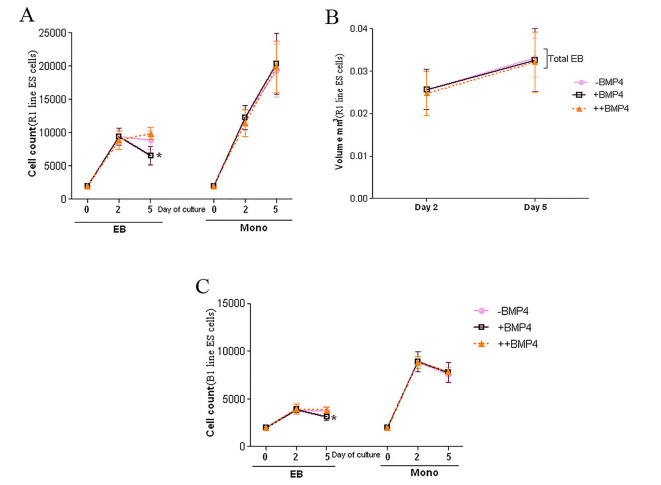
Analyses of cell count and volume in EB and monolayer culture system. (A) The cell count of EB and monolayer culture conditions after 2 and 5 days of culturing R1 cell lines in different experimental conditions. (B) The comparison of the total volume of the R1 EBs cultured in different experimental conditions. (C) The cell count of EB and monolayer culture conditions after 2 and 5 days of culturing B1 cell lines in different experimental conditions. Data are expressed as mean±SD (*P<0.05).

**Figure 3:**
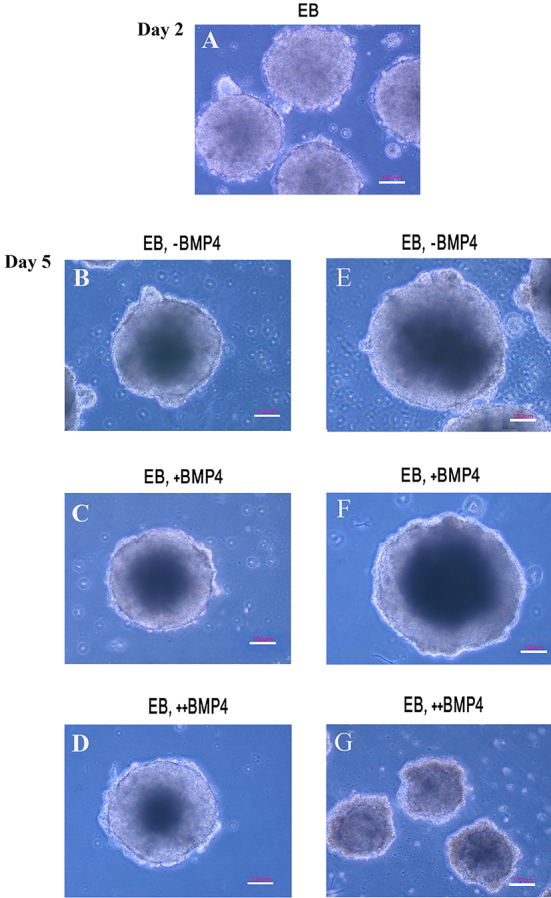
Phase contrast microscopy of EB culture method in (A-D) R1 cell line on day 2 and 5, (E-F) B1 cell line at 15000 cell/30 µL hanging drop and (G) 2000 cell/20 µL on day 5 of differentiation method. EB, Embryoid body; +BMP4, exposure after 2 days of culture; ++BMP4, exposure from the initiation of culture; -BMP4, control culture. Scale bar=100 µm.

To determine the effects of exposure time of BMP4 on ES cell population, we cultured two mouse ES cell lines (R1 and B1) in monolayer and EB culture system in two different periods of time and the cell count was performed on days 2 and 5. Viability assays of R1 cell line showed a significant reduction in the cell count of the EBs cultured in +BMP4 condition in comparison with ++BMP4 condition at day 5 (P=0.009). However, there was no significant difference in the cell number between EBs cultured in the presence of BMP4 compared to control condition at day 2 (P=0.60; figure 2A). Estimation of total volumes of EBs was performed at days 2 and 5 (figure 2B). The total volumes of the EBs were similar at days 2 (P=0.62) and 5 (P=0.76). Cavitation was started to form in the EBs at day 3. On day 5, all EBs (R1 line) had their own cavities. As shown in figure 3A-D, the cavity of the EB cultured in +BMP4 condition appeared darker than those cultured in the absence of BMP4, and the cavity of the EB in ++BMP4 was morphologically smaller than those in other groups with the same light intensity and magnification. Notably, in the EBs cultured in +BMP4 condition, the necrotic area appeared to be similar in size to those of control condition, but the cell count revealed a significant decrease in +BMP4 condition compared to control (approximately 26%; P=0.028).

Microscopic observations showed that B1 ES cell line failed to form cavity when treated by all experimental conditions in EB formation (figure 3G). Surprisingly, the data obtained from the cell count of B1 cell line was in agreement with the results obtained from those of R1 cell line. The cell number in the EBs cultured in +BMP4 condition showed a significant reduction in comparison with those of ++BMP4 condition at day 5 (P=0.009; figure 2C). Also, the total volume of EBs derived from B1 cells was smaller than those derived from R1 cell line cultured at the same condition with a significant decrease in cell number (P=0.009). The larger number of seeded B1 cells in hanging drop (15000 cell/30µl against 2000 cell/20µL) led to the formation of EBs with a similar cavity size to those from R1 cell line (figure 3E and F).

Cell count was also performed in monolayer culture system in R1 line. The number of viable cells in various conditions (with or without BMP4) of monolayer culture system did not show any significant difference in days 2 and 5 (P=0.34 and P=0.91, respectively). However, cell number showed significant increase in day 5 compared to day 2 (P<0.0001; figure 2A).

Similar to R1 line, cell count revealed no significant difference in B1 monolayer culture system at days 2 and 5 (P=0.91 and P=0.71, respectively), although there was a significant decrease between days 2 and 5 (P=0.005; figure 2C). In addition, the cells from B1 monolayer culture system showed a significant decrease in cell number than those derived from R1 cell line (P=0.009).

## Discussion

The present study showed that cell *counting* and determining viability of EBs influenced by BMP4 exposure in time-dependent manner. Supplementation of the EB cultured with BMP4 48 h after the beginning of the culture (late exposure), reduced cell number efficiently and showed greater cavity size than EB cultured in the presence of BMP4 from the beginning of culture (early exposure). Embryoid body appears to be a potent in vitro model to study signals for programmed cell death and cell survival during cavitation in mammals.^8^^,^^9^ In this three-dimensional spherical cell mass structure, enhancement of cell-*cell* interactions can *play an important role* in cell behavior.^10^ Embryoid body contain an outer layer of primitive endodermal cells that produced BMP2 and inner core of ectodermal cell that produced BMP4 after 3 days of culture.^9^ BMP2/4 signaling pathways promote differentiation of primitive endoderm to visceral endoderm and then can induce cavitation/programmed cell death in EB similar to proamniotic cavity formation in embryo. Notably, blocking of BMP4 signaling prevented apoptosis and there upon cavitation of EBs.^7^ Interestingly, the presence of exogenous BMP4 could enhance both endogenous BMP4 and Smad1 levels in EB differentiation method.^19^ In addition, previous studies have demonstrated that BMP4 can initiate the formation of the cavity in embryonic coelom through the development of visceral endoderm that can stimulate apoptotic cell death in early post-implantation mouse embryo.^3^ The outer layer of primitive endodermal cells in EBs also secretes a thick layer of basement membrane *known as* Reichert’s membrane. This is seen as a dark layer separating the endoderm from the undifferentiated core cells.^8^^,^^20^ Secretion of basement membrane by endodermal cells also produces survival signal for only outer ectoderm located adjacent to it and promotes formation of polarized columnar epithelium, while those in the centre die by apoptosis.^21^^,^^22^

In the EBs with small cavity at the center and cultured in ++BMP4 condition, an increase in both cell viability and number was observed. It can be assumed that culturing of the EBs in the presence of BMP4 for a longer period of time (from the beginning of EB formation) result in early cell differentiation which might fail the formation of functional visceral endodern produced BMP2 that can induce cavitation/programmed cell death in EBs.^23^ Therefore, exposure time of BMP4 can affect cell number in EBs. Other investigations also showed the time dependent impacts of BMP4 toward particular cell lineages in various ES cell differentiation protocols. For instance, long-term exposure to BMP4 is needed to differentiate ES cells toward hematopoietic cells; in contrast, short-term treatment could promote induction of cardiac differentiation.^11^^,^^12^

Moreover, the cell counts reduced in B1 cell line by +BMP4 administration in EBs. However, in contrast to the R1 cell line, the cavity formation failed as indicated with inverted microscopic observation. Different behaviours of various cell lines under the same experimental conditions have been shown previously.^14^ Similarly, serial sections of the EBs derived from S2 embryonal carcinoma cell line cultured in the presence of BMP4 revealed more small cavities located near the periphery of the EBs due to the cell death.^7^ Our data also indicated that seeding a higher cell density in hanging drops also led to the formation of a central cavity in B1. The cell density in hanging drop plays an important role in controlling the fate of ES cells.^24^

In addition, in the monolayer culture system cultured in ++BMP4 or +BMP4 condition, no change was observed in both cell viability and number. The cell number also was higher compared to EB formation. In line with our result, a comparison between monolayer culture systems and EBs showed that monolayer culture systems led to quick proliferation and formed multiple layers and it produced higher cells for subsequent analysis.^25^ Although exposure of the cells with BMP4 inhibited proliferation and induced apoptosis of the cells dose dependently, while higher concentrations of BMP decreased cellular viability to lower levels.^26^^,^^27^ Also, BMP4 exposure of ES cells induced apoptosis in mouse ES cell-derived neural precursors.^28^ The effects of growth factors were shown to depend on the cell culture procedure, concentration of factor or cell type. In monolayer culture system minimizes cell-cell contact and interaction, which consequently reduces intercellular signaling.^10^ It seems cell proliferation and viability rate may be affected by cell–cell interaction.

## Conclusion

Collectively, we show that BMP4 exposure time exerts different roles in cell number in EB formation process. Although, the role of BMP4 in cell death has been shown previously, our data reveal short-term BMP4 treatment induced EB cavitation may be due to decrease in cell viability. The data introduce a suitable procedure for the evaluation of programmed cell death in any study focused on the impact of different agents on apoptosis by EB cavity formation. Furthermore, cell viability depends on the cell lines and chosen culture protocol (EB versus monolayer). 

## References

[1] Chen D, Zhao M, Mundy GR (2004). Bone morphogenetic proteins. Growth Factors.

[2] Childs AJ, Kinnell HL, Collins CS, Hogg K, Bayne RA, Green SJ (2010). BMP signaling in the human fetal ovary is developmentally regulated and promotes primordial germ cell apoptosis. Stem Cells.

[3] O’Shea KS (2004). Self-renewal vs. differentiation of mouse embryonic stem cells. Biol Reprod.

[4] Yasuda E, Seki Y, Higuchi T, Nakashima F, Noda T, Kurosawa H (2009). Development of cystic embryoid bodies with visceral yolk-sac-like structures from mouse embryonic stem cells using low-adherence 96-well plate. J Biosci Bioeng.

[5] Downing GJ, Battey JF Jr (2004). Technical assessment of the first 20 years of research using mouse embryonic stem cell lines. Stem Cells.

[6] Khoo ML, McQuade LR, Smith MS, Lees JG, Sidhu KS, Tuch BE (2005). Growth and differentiation of embryoid bodies derived from human embryonic stem cells: effect of glucose and basic fibroblast growth factor. Biol Reprod.

[7] Coucouvanis E, Martin GR (1999). BMP signaling plays a role in visceral endoderm differentiation and cavitation in the early mouse embryo. Development.

[8] Hernández-García D, Castro-Obregón S, Gómez-López S, Valencia C, Covarrubias L (2008). Cell death activation during cavitation of embryoid bodies is mediated by hydrogen peroxide. Exp Cell Res.

[9] Qu X, Zou Z, Sun Q, Luby-Phelps K, Cheng P, Hogan RN (2007). Autophagy gene-dependent clearance of apoptotic cells during embryonic development. Cell.

[10] Keller G (2005). Embryonic stem cell differentiation: emergence of a new era in biology and medicine. Genes Dev.

[11] Zhang P, Li J, Tan Z, Wang C, Liu T, Chen L (2008). Short-term BMP-4 treatment initiates mesoderm induction in human embryonic stem cells. Blood.

[12] Pick M, Azzola L, Mossman A, Stanley EG, Elefanty AG (2007). Differentiation of human embryonic stem cells in serum-free medium reveals distinct roles for bone morphogenetic protein 4, vascular endothelial growth factor, stem cell factor, and fibroblast growth factor 2 in hematopoiesis. Stem Cells.

[13] Young JC, Dias VL, Loveland KL (2010). Defining the window of germline genesis in vitro from murine embryonic stem cells. Biol Reprod.

[14] Kim SE, Kim BK, Gil JE, Kim SK, Kim JH (2007). Comparative analysis of the developmental competence of three human embryonic stem cell lines in vitro. Mol Cells.

[15] West FD, Roche-Rios MI, Abraham S, Rao RR, Natrajan MS, Bacanamwo M (2010). KIT ligand and bone morphogenetic protein signaling enhances human embryonic stem cell to germ-like cell differentiation. Hum Reprod.

[16] Pucéat M (2007). TGFbeta in the differentiation of embryonic stem cells. Cardiovasc Res.

[17] Nagy A, Rossant J, Nagy R, Abramow-Newerly W, Roder JC (1993). Derivation of completely cell culture-derived mice from early-passage embryonic stem cells. Proc Natl Acad Sci U S A.

[18] Baharvand H, Matthaei KI (2004). Culture condition difference for establishment of new embryonic stem cell lines from the C57BL/6 and BALB/c mouse strains. In Vitro Cell Dev Biol Anim.

[19] Adelman CA, Chattopadhyay S, Bieker JJ (2002). The BMP/BMPR/Smad pathway directs expression of the erythroid-specific EKLF and GATA1 transcription factors during embryoid body differentiation in serum-free media. Development.

[20] Fagundez CB, Loresi MA, Ojea Quintana ME, Delcourt SM, Testa R, Gogorza SJ (2009). A simple approach for mouse embryonic stem cells isolation and differentiation inducing embryoid body formation. Cell Biol Int.

[21] Murray P, Edgar D (2000). Regulation of programmed cell death by basement membranes in embryonic development. J Cell Biol.

[22] Abud HE (2004). Shaping developing tissues by apoptosis. Cell Death Differ.

[23] Fujiwara T, Dunn NR, Hogan BL (2001). Bone morphogenetic protein 4 in the extraembryonic mesoderm is required for allantois development and the localization and survival of primordial germ cells in the mouse. Proc Natl Acad Sci U S A.

[24] Wobus AM, Boheler KR (2005). Embryonic stem cells: prospects for developmental biology and cell therapy. Physiol Rev.

[25] Tilgner K, Atkinson SP, Golebiewska A, Stojkovic M, Lako M, Armstrong L (2008). Isolation of primordial germ cells from differentiating human embryonic stem cells. Stem Cells.

[26] Cate HS, Sabo JK, Merlo D, Kemper D, Aumann TD, Robinson J (2010). Modulation of bone morphogenic protein signalling alters numbers of astrocytes and oligodendroglia in the subventricular zone during cuprizone-induced demyelination. J Neurochem.

[27] Hjertner O, Hjorth-Hansen H, Börset M, Seidel C, Waage A, Sundan A (2001). Bone morphogenetic protein-4 inhibits proliferation and induces apoptosis of multiple myeloma cells. Blood.

[28] Gambaro K, Aberdam E, Virolle T, Aberdam D, Rouleau M (2006). BMP-4 induces a Smad-dependent apoptotic cell death of mouse embryonic stem cell-derived neural precursors. Cell Death Differ.

